# Overall diet quality and risk of recurrence and progression of non-gallstone-related acute pancreatitis: a prospective cohort study

**DOI:** 10.1007/s00394-017-1526-8

**Published:** 2017-08-30

**Authors:** Viktor Oskarsson, Omid Sadr-Azodi, Andrea Discacciati, Nicola Orsini, Alicja Wolk

**Affiliations:** 1 Institute of Environmental Medicine, Karolinska Institutet, Nobels väg 13, Box 210, 171 77 Stockholm, Sweden; 2 Centre for Clinical Research Sörmland, Uppsala University, 631 88 Eskilstuna, Sweden; 3 Department of Public Health Sciences, Karolinska Institutet, 171 77 Stockholm, Sweden

**Keywords:** Pancreatitis, Secondary prevention, Diet, Epidemiology, Prospective studies

## Abstract

**Purpose:**

An incident episode of acute pancreatitis is often followed by recurrent attacks and/or progression to chronic pancreatitis, especially if the etiology is non-gallstone-related. We examined whether overall diet quality influences the natural history of non-gallstone-related acute pancreatitis.

**Methods:**

Three hundred and eighty-six individuals (born 1914–1952) were included in a prospective study, all of whom had an incident diagnosis of non-gallstone-related acute pancreatitis in the Swedish National Patient Register between 1998 and 2013. Participants were already enrolled in two population-based cohorts and had completed a food frequency questionnaire in 1997. Overall diet quality was calculated using a recommended food score (RFS), which was based on 25 food items. Post-diagnosis follow-up was conducted throughout 2014 for recurrence of acute pancreatitis and/or progression to chronic pancreatic disease (including cancer). Hazard ratios were estimated using Cox models.

**Results:**

During 1859 person-years of follow-up, 23.3% of the study population (*n* = 90) developed recurrent or progressive pancreatic disease. An inverse association was observed between the RFS and risk of recurrent and progressive pancreatic disease after adjustment for age and sex (hazard ratio for each 2-unit increase 0.90, 95% confidence interval 0.81–1.01) (*P*_overall association_ = 0.06). However, the association became weaker and was not statistically significant after adjustment for other potential confounders, including alcohol drinking and cigarette smoking (*P*_overall association_ = 0.27).

**Conclusions:**

In this prospective study of individuals with non-gallstone-related acute pancreatitis, there was no clear association between overall diet quality and risk of recurrent and progressive pancreatic disease.

## Introduction

Acute pancreatitis is an inflammatory disorder of the pancreas, which, in a majority of patients, has a benign and self-limiting course [1]. However, as meta-regressed by Sankaran et al. [2], it is often followed by recurrent attacks (22%) and/or progression to a chronic disease state, so-called chronic pancreatitis (10%). Even though its natural history has been extensively studied over the last decades, the risk factors that might affect the transition from incident (first attack) to recurrent and progressive acute pancreatitis are largely unknown, especially those that may be protective and modifiable. In more recent studies [3–9], the most consistent associations have been seen for a clinical-etiological classification of alcohol-related etiology [5–7], a severe disease course [7–9], and a cigarette smoking habit [5–8] (all positive in direction).

In a highly cited review article from 2013 by Yadav and Lowenfels [10], the role of diet in acute and chronic pancreatitis was quoted as “an important area for future research”. Since then, however, only six larger studies have been published on the association between dietary factors and incidence of acute pancreatitis [11–16], of which three were conducted by our research group using non-gallstone-related acute pancreatitis as the outcome [11–13]. Furthermore, to our knowledge, there are no studies on the association between dietary factors and risk of recurrent and progressive pancreatic disease among individuals with acute pancreatitis.

Therefore, using data from two large prospective cohorts, the Swedish Mammography Cohort (SMC) and the Cohort of Swedish Men (COSM), we examined whether overall diet quality was associated with recurrence and progression of non-gallstone-related acute pancreatitis.

## Methods

### Study participants

The enrollment of participants in the SMC took place between 1987 and 1990. A total of 66,651 women (born 1914–1948) were recruited from central Sweden (Uppsala and Västmanland counties). At baseline, all women answered a questionnaire about diet and lifestyle habits. Subsequent questionnaires were sent in 1997 and in 2009, to which 39,227 and 25,332 of the original women answered. The enrollment of participants in the COSM took place in 1997. A total of 48,850 men (born 1918–1952) were recruited from central Sweden (Örebro and Västmanland counties). At baseline, all men answered an identical questionnaire to that answered by women (aside for sex-specific questions) and 26,156 of them answered the subsequent questionnaire in 2009. Since the first female questionnaire did not contain questions on a number of important co-variables, including cigarette smoking and physical activity, we restricted the present study to questionnaire data collected in 1997 (main analysis) and in 2009 (sensitivity analyses). A detailed description of the two cohorts, as well as a full version of each questionnaire, is available at http://www.ki.se/en/imm/unit-of-nutritional-epidemiology. Approval for the study was given by the Regional Ethical Board at Karolinska Institutet (Stockholm, Sweden) (2010/1091-31/1 and 2014/2032-32).

### Study variables

Usual dietary intake over the previous year was assessed by the participants using a 96-item food frequency questionnaire (FFQ) in 1997 and a 132-item FFQ in 2009. For most foods, there were eight possible frequency-of-consumption responses, which ranged from “never” to “3 or more times/day”. For commonly consumed foods, such as milk and bread, there were instead open-ended responses.

Overall diet quality was calculated using a recommended food score (RFS), which, in 1997, included fruits (*n* = 4), vegetables (*n* = 11), legumes (*n* = 1), nuts (*n* = 1), low-fat dairy products (*n* = 2), whole grains (*n* = 3), and fish (*n* = 3) (see Table 2, footnotes, for individual foods). The concept of a RFS was originally developed by Kant et al. [17] and has been adapted to the FFQ in our study [18, 19]. It is calculated by summing the foods that are consumed at least weekly (adding up to a maximum score of 25). In addition, we calculated a non-RFS to account for the weekly consumption of less healthy foods (*n* = 21; see Table 2, footnotes, for included foods) [18]. The FFQ in 1997 has been validated for nutrient intakes by comparing it with multiple 24-h recall interviews, with a mean correlation of 0.7 for macronutrients [20]. It has also been validated for some of the individual components of the RFS, including vegetable and fruit items (correlation = 0.4–0.7) [21], fish items (correlation = 0.4–0.5) [22], and dairy items (correlation = 0.4–0.7) [23].

On each questionnaire, the participants reported on their sociodemographic factors (e.g., age and education), anthropometric measurements (e.g., weight and height), lifestyle habits (e.g., cigarette smoking and alcohol drinking), medical history of selected diseases (e.g., diabetes and hyperlipidemia), and use of selected drugs (e.g., postmenopausal hormones and aspirin). Complementary data on their medical history were obtained via linkage to, amongst others, the Swedish National Patient Register (SNPR) and the Swedish National Diabetes Register.

### Study cohort and follow-up

There were 917 individuals (557 men and 360 women) who had a diagnosis of acute pancreatitis in the SNPR between 1998 and 2013 [diagnosis code K85 in International Classification of Diseases (ICD)-10]. In this register, which has had an almost complete national coverage of in-hospital care since 1987 and of out-hospital specialist care since 2001 [24], the diagnosis code of acute pancreatitis (primary and secondary) has been shown to have a high validity [positive predictive value between 83% (definitive disease) and 98% (probable disease)] [25]. Individuals were excluded if their personal identity numbers were incorrect, if their energy intakes were extreme (>3 standard deviations of the sex-specific log-transformed mean), or if their medical history included prior pancreatic disease [including cancer, for which data were obtained from the Swedish National Cancer Registry (SNCR)] (diagnosis code 577 or 157 in ICD-8 and ICD-9 and K85, K86, K87, or C25 in ICD-10). (*n* = 114.) Individuals were also excluded if (1) there were indications of underlying chronic pancreatic disease (defined by diagnosis code K86, K87, or C25 in ICD-10) or symptomatic gallstone disease (defined by diagnosis code K85.1 or K80 in ICD-10 or by surgical code JKA20, JKA21, JKE00, JKE02, JKE12, JKE18, or JKB30 in NOMESCO Classification of Surgical Procedures) up to 90 days after the diagnosis (hereinafter referred to as a 90-day post-diagnosis investigation) or (2) a full 90-day post-diagnosis investigation was not “completed” [due to death or end of the recruitment period (death data were obtained from the Swedish National Cause of Death Register)]. (*n* = 417). The resulting study cohort consisted of 386 individuals (255 men and 131 women). The 90-day post-diagnosis window was to allow for a fairly detailed etiological investigation (as we were only interested in acute pancreatitis of non-gallstone-related etiology) and a complete resolution of the initial episode (as we were only interested in recurrence and progression, not in readmission). By definition, all acute pancreatitis-related hospital care that occurred within the first 90 days was considered to be part of the initial episode. While there has been no validation of the subtypes of acute pancreatitis in the SNPR, the percentage of all episodes that were classified as non-gallstone-related according to our definition was highly similar to that in Swedish studies with access to medical charts (54 vs. 52–61%) [7, 25, 26].

Information on recurrent episodes of acute pancreatitis was obtained from the SNPR until the end of 2014 (diagnosis code K85 in ICD-10). Since the previous validation study on the diagnosis of acute pancreatitis was restricted to incident episodes (Rickard Ljung, personal communication, 2014) [25], we only used primary diagnosis codes for specificity reasons. Information on other pancreatic diseases was obtained from the SNPR and the SNCR (diagnosis code K86, K87, or C25 in ICD-10).

### Statistical analyses

We compared the characteristics of the study cohort with those of the entire baseline cohort (i.e., all eligible participants in the SMC and the COSM) as well as according to levels of the RFS.

Cox regression models, with attained age as time-scale, were used to calculate hazard ratios (HRs) and 95% confidence intervals (CIs). Individuals contributed person-years from the start of follow-up (i.e., 90 days after the incident episode) until the diagnosis of recurrent acute pancreatitis, other pancreatic diseases, death, or end of follow-up on 31 December 2014. Combining acute pancreatitis and the other pancreatic diseases into a composite failure variable gave similar results to those using acute pancreatitis as a single failure variable. Therefore, particularly with respect to the statistical power of the Cox regression, the main outcome of this study was the combination of recurrent and progressive pancreatic disease. In addition, to keep the number of events per parameter as high as possible [27], we (1) modeled categorical co-variables using binary indicators and continuous co-variables assuming linearity and (2) handled missing data using multiple imputation by chained equations (a total of 40 data sets were created and combined using Rubin’s rule) (see Table 1, footnotes, for proportions of missing data) [28].

**Table 1:** Baseline (1998) characteristics of the entire baseline cohort and the study cohort that developed non-gallstone-related acute pancreatitis during the recruitment period (1998–2013)

Baseline characteristics^a,b^ (1998)	Baseline cohort	Study cohort
	(*n* = 81,360)	(*n* = 386)
Male sex (%)	55.2	66.6
Age (mean, years)	60.9	62.4
University education (%)	17.5	14.6
Body mass index (mean, kg/m^2^)	25.4	26.5
Physical activity ≥1 h of exercise/week (%)	79.1	74.2
Ever use of postmenopausal hormones (%)^c^	56.0	70.0
History of diabetes (%)	7.7	7.8
History of hyperlipidemia (%)	13.0	14.7
Current smoker (%)	22.3	27.6
Pack-years smoked (mean)	23.1	23.9
Current drinker (%)	84.2	83.2
Alcohol intake (mean, g/week)	82.5	97.1
Dietary intakes (mean)		
Recommended food score^d^	10.9	10.4
Non-recommended food score^d^	8.1	7.9
Energy (kcal/day)	2246	2237

The baseline cohort consisted of participants in the Swedish Mammography Cohort and the Cohort of Swedish Men who were free of pancreatic disease and cancer and who had plausible energy intakes (≤3 standard deviations of the sex-specific log-transformed mean)

^a^Characteristics of the study cohort were standardized to the sex and age distribution (<55, 55–64, 65–74, and >74 years) of the baseline cohort (except for age and sex, which were standardized for each other)

^b^Characteristics were based on complete data only. The percentages of missing data in the baseline cohort and the study cohort were as follows: 0.4 and 0.3% for education, 3.6 and 5.4% for body mass index, 10.6 and 9.8% for exercise, 1.2 and 0.0% for postmenopausal hormones, 1.6 and 1.8% for smoking status, 0.7 and 1.0% for alcohol status, 4.3 and 3.4% for the recommended food score, and 3.1 and 3.1% for the non-recommended food score

^c^Among postmenopausal women only

^d^The maximum value is 25 for the recommended food score and 21 for the non-recommended food score

The RFS was modeled as a continuous variable assuming linearity (i.e., a constant change in risk for each unit of change in exposure). To relax this assumption, we modeled the RFS using a 3-knot restricted cubic spline in the continuous analysis (knots at the 10th, 50th, and 90th percentile of its distribution) [29] and also according to approximate tertiles in a separate categorical analysis. The standard assumption of proportional hazards along the time-scale was evaluated by modeling the possible interaction between the time-scale and the RFS [30]; no violation of proportionality was detected.

Multivariable models were adjusted for sex, education (≤12, >12 years), smoking status (never/former smoker, current smoker), body mass index (calculated as weight divided by height squared; continuous, kg/m^2^), physical activity (<1, ≥1 h of exercise/week), history of diabetes (no, yes) and hyperlipidemia (no, yes), alcohol drinking status (never/past drinker, current drinker), the non-RFS (continuous, score), and energy intake (continuous, kcal/day) (all assessed at baseline) as well as length of hospital stay (used as a proxy for disease severity; continuous, days) and calendar year at diagnosis (continuous). In a sensitivity analysis, which aimed to account for potential residual confounding, we included an extra category of current smokers (<30, ≥30 pack-years smoked) and current drinkers (<24, ≥24 g of alcohol/day) and another modality of physical activity [walking or cycling (<20, ≥20 min/day)]. For the same reason, we also relaxed the linearity assumption of the continuous co-variables (using 3-knot restricted cubic splines). For comparison with the multiple imputed results, a complete-case analysis was performed as another sensitivity analysis [31].

To assess potential changes in dietary intake following a diagnosis of non-gallstone-related acute pancreatitis, as well as changes in cigarette smoking and alcohol drinking, we analysed two subsets of the data. One consisted of those who had a diagnosis between 1998 and mid-2009 and who, thereafter, had answered the questionnaire in 2009, while the other consisted of all eligible participants in the SMC and the COSM who had answered the questionnaire in 2009.

Statistical significance was set at a two-sided *P* value less than 0.05. Analyses were performed using Stata version 12.1 (StataCorp, College Station, TX, USA).

## Results

A total of 386 individuals (255 men and 131 women) were included in this study, all of whom had an incident diagnosis of non-gallstone-related acute pancreatitis between 1998 and 2013. The mean age at diagnosis was 70.9 years (standard deviation 9.5 years) and the median length of hospital stay was 4 days (interquartile range 3–8 days). The baseline characteristics of the study population are shown in Table 1, along with those of everyone who were eligible for study inclusion. These data point to well-known (or suggested) positive associations of acute pancreatitis, such as those with male sex, smoking, obesity, hyperlipidemia, and use of postmenopausal hormones. In contrast, the difference in alcohol intake was somewhat smaller than expected [mean difference 14.5 g/week (31.4 g in men and 0.0 g in women)] and no clear association was observed with diabetes.

The study population was followed up for a mean of 4.8 years (a total of 1859 person-years of follow-up), and during this time, 90 cases (23.3%) of recurrent and progressive pancreatic disease were recorded. Of these, 76 had recurrent acute pancreatitis, 11 had non-malignant chronic disease, and 3 had cancer. Even though the male (*n* = 60)-to-female (*n* = 30) case ratio was 2:1, the disease rate was highly similar in both sexes (5.1 cases and 4.4 cases, respectively, per 100 person-years). The distribution of diagnosis and baseline characteristics according to the RFS is given in Table 2. Individuals who scored higher on the RFS tended to be women, more educated, more physically active, and less likely to smoke. These individuals were also more likely to have a history of diabetes and hyperlipidemia as well as to drink alcohol, although they did so with greater moderation. Higher levels of the RFS were associated with higher levels of energy intake and the non-RFS (Spearman correlation = 0.2 and 0.3, respectively).

**Table 2:** Characteristics of the study cohort (*n* = 386 individuals with non-gallstone-related acute pancreatitis) according to approximate tertiles of the recommended food score

	Recommended food score (median)^a^		
	<9 (6)	9–12 (10)	>12 (14)
Characteristics at diagnosis (1998–2013)^b^			
Male sex (%)	76.5	62.7	59.7
Age (mean, years)	70.0	71.4	70.9
Length of hospital stay (median, days)^c^	4.5	4.5	4.9
Characteristics at baseline (1998)^b^			
University education (%)	11.9	14.5	15.8
Body mass index (mean, kg/m^2^)	26.2	26.6	26.7
Physical activity ≥1 h of exercise/week (%)	64.7	79.1	82.4
History of diabetes (%)	7.3	7.4	8.7
History of hyperlipidemia (%)	14.5	16.2	17.4
Current smoker (%)	39.0	20.8	19.6
Pack-years smoked (mean)	24.1	27.9	26.9
Current alcohol drinker, %	80.4	88.0	85.7
Alcohol intake (mean, g/week)	125.0	83.6	93.7
Dietary intakes (mean)			
Recommended food score^a^	5.7	10.4	14.8
Non-recommended food score^d^	7.2	7.7	9.2
Energy (kcal/day)	2082	2259	2647

^a^The recommended food score includes fruit (apples and pears; bananas; citrus fruits; and berries), vegetables (spinach; lettuce and green salad; cabbage; cauliflower; broccoli and Brussels sprouts; carrots; beetroots; tomatoes and tomato juice; sweet pepper; green peas; and mixed vegetables), legumes, nuts, low-fat dairy products (reduced-fat milk; reduced-fat cultured milk and yogurt), whole grains (whole grain bread; crisp and hard bread; and oatmeal), and fish (herring and mackerel; salmon, whitefish, and char; and cod, saithe, and fish fingers)

^b^Continuous characteristics were standardized to the sex and age distribution (<70, ≥70 years) of the study cohort, whereas categorical characteristics were only standardized to the sex distribution because of zero observations in some of the age-specific strata

^c^Used as a proxy for disease severity

^d^The non-recommended food score includes red meat (minced meat; pork; beef and veal; and liver and kidney), processed meat (sausage and hot dogs; ham, salami, and processed meat cuts; liver paté; and blood sausage), fried potatoes, French fries, potato crisps, solid fats (butter and margarines), whole-fat milk products (full-fat milk; cheese; ice cream; and cream), white bread, refined cereals (spaghetti and macaroni), sugar, sweets, and buns and cookies

In age- and sex-adjusted Cox regression analyses, we observed a borderline statistically significant inverse association between the RFS and risk of recurrent and progressive pancreatic disease (Table 3, model 1). The HR for each 2-unit increase in the RFS (equal to 0.5 standard deviations of its distribution and corresponding to a weekly consumption of two healthy foods) was 0.90 (95% CI 0.81–1.01) in the continuous analysis (*P*_overall association_ = 0.06), with no evidence of departure from the assumption of linearity (*P*_non-linearity_ = 0.91). Similarly, in the categorical analysis, the HR was 0.59 (95% CI 0.34–1.00) for the highest compared with the lowest tertile (*P*_overall association_ = 0.14). Further adjustment for other dietary factors (i.e., energy intake and the non-RFS) did not appreciably change the results (data not shown). However, the inverse association became weaker and was not statistically significant after further adjustment for the other potential confounders (Table 3, model 2). The multivariable-adjusted HR for each 2-unit increase in the RFS was 0.92 (95% CI 0.81–1.06) in the continuous analysis (*P*_overall association_ = 0.27), and it was 0.69 (95% CI 0.36–1.29) for the comparison of extreme tertiles in the categorical analysis (*P*_overall association_ = 0.45).

**Table 3:** Cox regression-derived hazard ratios of recurrent and progressive pancreatic disease, based on multiple imputed data and according to the recommended food score

Recommended food score	No. of participants	No. of cases	No. of person-years	Hazard ratio (95% CI)	
				Model 1^a^	Model 2^b^
Continuous model					
Per 2-unit increase	386	90	1859	0.90 (0.81–1.01)	0.92 (0.81–1.06)
*P* for non-linearity^c^				0.91	0.78
Categorical model^d^					
<9	125	36	556	1.00 (ref)	1.00 (ref)
9–12	126	29	556	0.88 (0.53–1.46)	0.94 (0.53–1.65)
>12	122	24	692	0.59 (0.34–1.00)	0.69 (0.36–1.29)
*P* for overall association^e^				0.14	0.45

*CI* confidence interval

^a^Adjusted for attained age (time-scale) and sex

^b^Adjusted for attained age (time-scale), sex, education (≤12, >12 years), smoking status (never/former smoker, current smoker), body mass index (continuous, kg/m^2^), physical activity (<1, ≥1 h of exercise/week), history of diabetes (no, yes) and hyperlipidemia (no, yes), alcohol intake (never/past drinker, current drinker), the non-recommended food score (continuous, score), and energy intake (continuous, kcal/day) (all assessed at baseline) as well as length of hospital stay (continuous, days) and calendar year of diagnosis (continuous)

^c^Test for non-linearity was calculated using the Wald test in a restricted cubic spline model, which tested the coefficient of the second spline transformation equal to zero

^d^Values on the number of participants, cases, and person-years for the categorical model do not sum up to the entire study cohort because of different exposure distributions across the imputed data sets (*n* = 40). Reported values are based on the participants who had complete data on the recommended food score

^e^Test for overall association was calculated using the Wald test, which tested the coefficients of the categorical variable jointly equal to zero

Restricting the outcome to recurrent episodes of acute pancreatitis did not substantially change the exposure–outcome association. As an example, the multivariable-adjusted HR for each 2-unit increase in the RFS was 0.94 (95% CI 0.81–1.09) in the continuous analysis. The results were also comparable in the sensitivity analysis in which we performed (1) a more detailed adjustment for potential confounders (multivariable-adjusted HR 0.90, 95% CI 0.78–1.04) and (2) a complete-case analysis (*n* = 307 including 77 cases) (multivariable-adjusted HR 0.92, 95% CI 0.79–1.06). Finally, similar to the findings for the RFS, we observed no statistically significant association between the non-RFS and risk of recurrent and progressive pancreatic disease (multivariable-adjusted HR for each 2-unit increase in the non-RFS 1.04, 95% CI 0.88–1.24).

To examine whether there were any post-diagnosis changes in dietary intake, for which purpose the RFS was categorized into approximate quartiles, we used data from 139 men and women who had a diagnosis of non-gallstone-related acute pancreatitis before they had answered the questionnaire in 2009. Up to 83.1% of them stayed in the same or an adjacent quartile of the RFS between the questionnaire in 1997 and that in 2009. Although the number of cases was small in this subset (*n* = 40), which widened the CIs, we found a similar exposure–outcome association with post-diagnosis RFS as with pre-diagnosis RFS (age- and sex-adjusted HR for each 2-unit increase in the post-diagnosis RFS 0.86, 95% CI 0.73–1.01). With respect to the other factors that were examined, that is, cigarette smoking and alcohol drinking, there were apparent changes between the questionnaires. Among those who had reported that they smoked cigarettes and drank alcohol in 1997, up to 73.3 and 17.5%, respectively, reported that they had stopped doing so by 2009. Since these changes, at least in theory, could reflect national trends over time rather than being secondary to a diagnosis of acute pancreatitis, we also used data from everyone who had been eligible for study inclusion, given that they had completed the questionnaire in 2009 (*n* = 46,538). After accounting for differences in sex distribution, the percentage who had stayed in the same or an adjacent quartile of the RFS (83.1 vs. 81.8%) and who had stopped smoking cigarettes (68.8 vs. 62.2%) was similar in the subsets with and without acute pancreatitis according to the questionnaires in 1997 and 2009—however, the percentage who had discontinued drinking alcohol was clearly larger among individuals with acute pancreatitis (17.8 vs. 7.5%). Moreover, among those who reported that they still drank alcohol in 2009, the alcohol intake had decreased far more in this subset (mean difference −24.0 g/week) than in that of all eligible participants (mean difference −5.8 g/week).

## Discussion

In this prospective cohort study of 386 individuals with non-gallstone-related acute pancreatitis, in whom the incident episode was diagnosed between 1998 and 2013, and who were followed up for a mean of 4.8 years, there was no clear association between overall diet quality and risk of recurrent and progressive pancreatic disease. An inverse association was observed with the RFS after adjustment for age and sex, albeit only borderline in statistical significance, but it became weaker and was not statistically significant after further adjustment for other potential confounders, including alcohol drinking and cigarette smoking.

Large epidemiologic data on the role of diet in acute pancreatitis range from sparse (incident episodes) to almost non-existent (recurrent and progressive episodes). Apart from a cross-sectional study from China that examined a high-meat dietary pattern (defined as “containing more than 50% of the flesh of animals”) [16], there are no studies on the association between dietary patterns and risk of acute pancreatitis. One advantage of studying dietary patterns is that they account for the whole diet, including interactions between foods with different nutrient contents. This is especially true compared to individual foods or nutrients, which only reflect a small piece, or a snapshot, so to speak, of the whole diet. The RFS is based on the weekly consumption of a variety of recommended foods (e.g., fruits, vegetables, and fish) and is an established, simple, and portion-size independent measure of overall diet quality [17]. Some of its individual components have already been studied in relation to risk of acute pancreatitis, as have other foods and nutrients. Our research group has observed associations between consumption of vegetables and fish (both inverse in direction) and high-glycemic load foods (positive in direction) and incidence of non-gallstone-related acute pancreatitis [11–13]. In turn, Prizment et al. examined some foods (*n* = 2) and several nutrients (*n* = 7) in the Iowa Women’s Health Study and they observed positive associations between total and saturated fat intake and incidence of acute pancreatitis [14]. More recently, Setiawan et al. used data from the Multiethnic Cohort Study to analyse the associations of nine foods and nine nutrients with incidence of acute pancreatitis [15]. While a number of dietary factors were associated with gallstone-related episodes (e.g., red meat, eggs, saturated fat, dietary fiber, and milk), only dietary fiber intake was associated with non-gallstone-related episodes (inverse in direction). The authors also analysed recurrent acute pancreatitis and chronic pancreatitis as a combined outcome, for which they observed a positive association with red meat consumption. However, as opposed to our study, those patients were not compared with acute pancreatitis patients without recurrence or progression but instead with pancreatitis-free individuals. Potential biological mechanisms to explain all of these associations have already been proposed and discussed [11–15], but, in brief, they may be due to direct effects (e.g., changes in inflammatory and oxidative stress markers or in pancreatic enzyme secretion) or indirect effects (e.g., changes in triglyceride levels or in prevalence of diabetes and obesity). Such mechanisms, if present, may also lead to a lower (or higher) risk of recurrent and progressive pancreatic disease among individuals with acute pancreatitis. However, it was not considered possible to examine individual foods and nutrients in our study. The number of events per parameter was low to begin with, which led to wide CIs in the Cox regression [27], and inclusion of multiple dietary items would have made that number even lower.

In more recent studies on the natural history of acute pancreatitis, the risk for recurrent attacks has ranged from 17.5 to 35.5% and the risk for chronic pancreatitis from 0.0 to 7.6% [3–9]. Although the risks in our study were within these ranges (19.7 and 2.8%, respectively), they are lower in a relative comparison, because individuals with gallstone-related acute pancreatitis were not included [i.e., the subtype with the lowest risk of recurrence (≈12%)]. There are several potential explanations for the somewhat lower risks in our data. First, the study population consisted of elderly men and women (mean age at diagnosis 70.9 years), who had a mostly light alcohol intake (mean intake 15.8 g/day, with only 6.9% of them drinking more than 40 g/day). Younger age [5, 6], as well as a clinical-etiological classification of alcohol-related etiology [5–7], has been positively associated with recurrence and progression in some of the previous studies. An alcohol-related etiology was there defined as a documented alcohol abuse or as a self-reported alcohol intake of at least 40–60 g/day. Second, in contrast to the previous studies, we used a 90-day post-diagnosis window to define the study population, during which (1) diagnoses of chronic pancreatic diseases were not allowed and (2) acute pancreatitis-related hospital care was considered to be part of the initial episode. The reason for this was to avoid inclusion of acute-on-chronic pancreatic disease (into the study population) and early readmissions of acute pancreatitis (into the composite failure variable). As an example, Ahmed Ali et al. observed that “[Chronic pancreatitis] was diagnosed within the first 3 months…in 6 (12%) patients. Such short interval suggests that this acute pancreatitis episode was the first manifestation of an existing [chronic pancreatitis]” [8]. Furthermore, it has been reported that less than 30% of early readmissions (i.e., within 30 days of discharge) are due to recurrent acute pancreatitis [32, 33], whereas later readmissions are more likely to be so [32]. Finally, we censored follow-up at the first diagnosis of any pancreatic disease, irrespective of it being recurrent or progressive. The other studies instead calculated separate risks for recurrent and progressive disease; and accordingly, the risk for progressive disease in our data was higher (6.5%) in a post hoc analysis that used that methodology.

An interesting and encouraging finding, especially from a clinical perspective, was that almost one-fifth of the study population had stopped drinking alcohol between 1997 and 2009, that is, before and after their incident episode of non-gallstone-related acute pancreatitis. In addition, the mean alcohol intake was reduced by 24.0 g/week among those who had continued to drink. This finding is not unique in itself as other studies have reported even higher discontinuation frequencies [4, 5, 8], but the setting in which it is observed is clearly unique—a prospective population-based cohort study. In contrast, the other studies recruited their participants in a hospital setting, with the specific purpose to study acute pancreatitis. As such, the physicians may have been extra motivated in their alcohol counseling (due to the planned research project) and the participants should have been highly motivated to participate in the study and to decrease their alcohol intake, which, in combination, might limit the generalizability of those results. Furthermore, the level of social desirability bias (i.e., systematic errors due to the desire of giving socially accepted answers) is expected to be extremely high with respect to alcohol intake in a pancreatitis-specific study. For these reasons, we believe that our study provides the most unbiased results to date on the effect of physicians’ alcohol counseling on the discontinuation of alcohol use.

A number of strengths and limitations need to be acknowledged. The prospective design, the inclusion of men and women, the rather long follow-up, and the ample data on diet and potential confounders were notable strengths. Apart from the small sample size, a major limitation was that diet (and most other factors) was assessed by self-reported questionnaires, which, inevitably, is associated with some degree of non-differential misclassification. In addition, there was only one assessment available for the majority of individuals (at baseline), with the possibility of changes during the follow-up period as well as following the diagnosis of non-gallstone-related acute pancreatitis. The RFS was, however, fairly stable over time (around 83% of the study population stayed in the same or an adjacent quartile of the RFS between 1997 and 2009). In addition, in a sensitivity analysis, the exposure–outcome association with post-diagnosis RFS was highly similar to that with pre-diagnosis RFS. It should be noted that we relied on register-based data to define the study population and to identify the outcomes of interest. However, the SNPR has been found to have a good validity for incident episodes of acute pancreatitis [25], and we have shown that the incidence rates in our cohorts were similar to those in Sweden as a whole [11–13]. A certain degree of misclassification of non-gallstone-related acute pancreatitis is still likely to have occurred (mainly due to misdiagnosis of very small gallstones, so-called microlithiasis) [34], even though the proportion of non-gallstone-related episodes in this study was highly similar to that in Swedish studies that relied on medical chart data [7, 25, 26]. Unfortunately, there has been no validation of recurrent acute pancreatitis or chronic pancreatitis in the SNPR. Any misclassification, whereby a hospitalization due to recurrent or progressive pancreatic disease was classified as due to another gastrointestinal disorder, and vice versa, should, however, have been unrelated to an individual’s overall diet quality. Nonetheless, to minimize the amount of false-positive cases, we only used primary diagnosis codes for the outcome variables. Another limitation was that the individuals had to “complete” a 90-day post-diagnosis investigation to be included in the study. Although this was done for a reason (i.e., as previously mentioned, to limit the influence of acute-on-chronic pancreatic disease and early readmissions of acute pancreatitis), the consequence was that we did not account for incident episodes that led to death or for recurrent episodes that happened very early. Not only does this left truncation hinder the study’s generalizability, it may also have led to bias and conservative risk estimates. Finally, the presence of unmeasured confounding (e.g., due to disease severity and treatment choices) or residual confounding (e.g., due to cigarette smoking and alcohol drinking) cannot be excluded, despite the fact that we adjusted for several baseline and diagnosis characteristics (including length of hospital stay, used as a crude proxy for disease severity). In addition to any residual confounding of cigarette smoking and alcohol drinking at baseline, there were apparent reductions in these habits during the follow-up period, which seemed to reflect both national trends (for cigarette smoking) [35] and post-diagnosis changes (for alcohol drinking). It is unclear how much, if at all, such misclassification might have confounded the association between pre-diagnosis RFS and risk of recurrent and progressive pancreatic disease. However, although it had a very low statistical precision, the association with post-diagnosis RFS did not appear to be confounded by post-diagnosis cigarette smoking and alcohol drinking in a sensitivity analysis (data not shown).

In summary, in this prospective cohort study of individuals with non-gallstone-related acute pancreatitis, there was no clear association between overall diet quality and risk of recurrent and progressive pancreatic disease, especially after adjustment for potential confounders. Because of the lack of epidemiologic data, further studies are needed to elucidate the potential role of diet in the natural history of acute pancreatitis.
